# Motivators and barriers towards clinical research participation: A population-based survey from an Arab MENA country

**DOI:** 10.1371/journal.pone.0270300

**Published:** 2022-06-24

**Authors:** Kamal M. Al-Shami, Wesam S. Ahmed, Karem H. Alzoubi

**Affiliations:** 1 Division of Tumor Metabolism and Microenvironment, German Cancer Research Center, Heidelberg, Germany; 2 Faculty of Biosciences, University of Heidelberg, Heidelberg, Germany; 3 Department of Drug Discovery and Development, Harrison School of Pharmacy, Auburn University, Auburn, AL, United States of America; 4 College of Health and Life Sciences, Hamad Bin Khalifa University, Qatar Foundation, Doha, Qatar; 5 Department of Clinical Pharmacy, Faculty of Pharmacy, Jordan University of Science and Technology, Irbid, Jordan; University of Oxford, UNITED KINGDOM

## Abstract

Jordan was the first Arab country to enact clinical research regulations. The country has a well-flourished pharmaceutical industry that leans heavily on clinical research (CR) for drug development and post-marketing surveillance. In this cross-sectional study, we sought to assess the public’s awareness and attitude towards CR as well as their perceived motivators and barriers to CR participation. A population-based, self-administered questionnaire was distributed to the general public in Jordan. Among the 1061 participants in this survey, 74% reported being aware of CR. The majority (70%) agreed to the role of CR in health promotion. Online information and healthcare staff were the two main sources of CR information for the participants. About 25% of the participants received prior invitations to participate in CR with 21% agreeing to participate. However, most participants of the current study (63%) were willing to participate in future CR. Contributing to science, benefiting others, and promoting one’s own health were the top motivating factors for participating in CR; while time constraints, fear of research procedure, and lack of interest were the most cited reasons for rejecting participation. Filling out questionnaire surveys, donating blood samples, and participating in physical examinations were the main CR contributions of the participants. Nearly 31% of the participants believed that CR is conducted in a responsible and ethical manner, while 57% did not have an opinion regarding the same matter. In addition, 49% and 44% were neutral with regards to the degree of harm and confidentiality posed by CR. While only 27% disagreed that CR exposes participants to some form of harm, 48% either strongly agreed (15%) or agreed (33%) that it maintains high level of confidentiality for participants. The current study provides insight into the public’s perception of CR in Jordan as well as its motivating factors and perceived barriers towards participating in CR. We envisage to utilize this insight as an aid in the design of vigilant future awareness campaigns and recruitment strategies.

## Introduction

Clinical research is the systematic approach intended to generate valuable knowledge for understanding diseases, promoting health, and diagnosing, preventing, and treating illnesses. This includes treatment, prevention, diagnostic and screening studies. It as well includes genetic studies, quality of life of patients, and epidemiological studies that seek to identify the patterns, causes, and control of disorders in groups of people [1–4]. This is opposed to public health research where the focus of the study is the population rather than the patients [5]. As such, human subjects’ enrollment into CR is considered a critical step in clinical studies [4]. However, suboptimal recruitment has long been a major challenge resulting in several negative consequences such as under-representative sample, decrease in statistical power, inconclusive results, and/or increase in research costs [6, 7]. Much of the general public remain unaware of CR and of the critical role their participation plays in advancing science [4]. Therefore, there is a need to assess the public’s awareness of CR and to understand participation determinants to identify effective interventional strategies that can improve voluntary participation of potential subjects and enhance participation retention.

Jordan, an Arab country which is also part of the Middle East and North Africa (MENA) region [8], is considered one of the most academically established countries in the Arab MENA region [9, 10]. Clinical research is not as common in Jordan, a developing country, as in the rest of the developed world [11]. In fact, CR in the whole Arab MENA region accounts for less than 1% of the global clinical trials [12]. However, Jordan was the first Arab country to enact clinical research regulations [13] and it is among the top countries in the Arab MENA region in the number of registered clinical studies per capita [14]. The country has witnessed a drastic population increase in the last few years due to millions of refugees coming from neighboring areas of conflict such Palestine, Syria, and Iraq [15, 16]. This population expansion has placed a great burden on the country’s healthcare system. As such, CR has become a fundamental tool to address these challenges and to allow for optimal healthcare services delivery. In addition, Jordan has a well-recognized pharmaceutical industry that exports around 80% of its production to more than 60 countries globally [17, 18]. As such, this pharmaceutical industry is considered the second largest exporting industry in the country [19]. This pharmaceutical sector leans heavily on CR for drug development and post-marketing surveillance, which further stresses and highlights the role of CR in the country.

Several studies have been carried out in the region [20–24] and internationally [25–31] to address CR enrollment facilitators and barriers. However, the findings from these studies may not be psychosocially relevant or generalizable to other populations with different socio-cultural determinants, especially when population-based studies from the region mainly included high-income countries such as Qatar, Saudi Arabia, Kuwait, UAE, and Oman [20–24]. For that matter, little is known about the general-public’s attitude and perceptions towards CR participation in a developing, non-high-income Arab MENA region country such as Jordan. Therefore, the aim of this study was to assess the population’s awareness and attitude to CR and to highlight their perceived facilitators and barriers to CR participation. This was achieved using a self-administered questionnaire and convenient sampling approach. We believe that results of this study can help in the design of informed CR enrollment strategies and enhancement of participants’ retention.

Current findings revealed that the Jordanian population is generally aware of CR, mostly because of the high literacy rate in the country. Participants of the current study expressed high willingness to participate in future clinical studies. However, only a small fraction of the general population has been approached to participate in clinical studies, suggesting that a modification to the current recruitment strategies is required to allow for more public engagement in research. Contributing to science and altruism were the most cited facilitators to research participation while time commitments, worries regarding the research process, and lack of interest were the main perceived barriers to participation. In addition, the study revealed a great lack of opinion when it comes to CR ethical conduct, confidentiality, and harm which seems to originate from lack of prior CR participation experience. More importantly, prior participation in CR was associated with positive attitudes towards CR and future participation, which suggests public engagement in clinical studies is an effective tool to promote positive attitudes to CR. These findings, along with the recommended recruitment interventions discussed here, considering the identified participation motivators and barriers, will greatly assist in the implementation of effective and informed recruitment strategies which we envisage to override current CR recruitment challenges in the country.

## Methods

### Study design

The paper-based questionnaire was carefully constructed by reviewing similar surveys from the region [20, 21, 30, 32, 33]. The survey utilized diverse question formats including multiple choice, “Yes” or “No”, multiple checkboxes, and Likert scale questions. The questionnaire consisted of four sections. The first section collected socio-demographic characteristics of participants including gender, age, nationality, marital status, education level, employment status, health insurance status, and chronic medical condition status. The second section assessed participants’ awareness of “clinical research”. The third section assessed participants’ motivators as well as their perceived barriers towards participating in CR. The last section assessed participants’ attitudes towards CR. Since Arabic is the native language of the country, the survey was translated from English to Arabic before deploying it to the public. Convenient sampling approach was used when sampling in the current study. Convenient sampling is the most commonly used non-probability sampling method that is based on approaching respondents who are “convenient” to the researcher, for instance by being located in reachable and accessible locations [34–36]. The study and questionnaire, both the Arabic and English versions, were approved by Jordan University of Science and Technology (JUST) Institutional Review Board (IRB) committee (Ref# 38/117/2018) with no consent form requirement.

### Study participants

The paper-based questionnaire survey was deployed to potential participants who met the selection criteria, which include an age of more than 18 years old, competency, and the ability to read and understand the Arabic language. Potentially eligible participants were conveniently approached, by trained research assistants and qualified graduate students, in public areas (markets, parks, universities, restaurants/cafeterias/coffee bars) and in different cities (Amman, Irbid, Zarqa, Mafraq, and Karak) to participate in this self-administered questionnaire. Informed verbal consent was obtained from participants after they were provided with a detailed description of the study as well as contact information should they decide to withdraw or have any concerns regarding the study. The participants were then provided a brief explanation of the meaning of clinical research and its scope. Responses were collected over a one-month period (between February and March 2019).

### Data analysis and figure preparation

Statistical Package for Social Science (SPSS®) V21 (SPSS Inc., Chicago, IL, USA) was used to analyze the data. Power analysis was carried out ensuring power is more than 80%. Descriptive statistics were utilized to summarize the data. All numerical information was reported as numbers and percentages out of the total responses with percentages approximated to zero decimal places in the main text. Chi-square test was used for cross-tabulation analysis of independent variables with the outcome variable of interest. A two-sided P-value of less than 0.05 was considered statistically significant. Figures were prepared using Microsoft Excel 13.

## Results

### Demographic characteristics

This self-administered, paper-based survey was directed towards the public in Jordan. About 1219 questionnaires were distributed, out of those 1165 were returned (response rate = 96%). Those with incomplete responses or that did not fulfill the eligibility criteria were excluded from analysis, leaving a total of 1061 surveys for analysis (effective rate 91%). There was an equal proportion of males to females (50%, n = 528 and 50%, n = 533 respectively) participants. Most participants were between 18 to 40 years of age (94%, n = 999), single (75%, n = 797), unemployed (66%, n = 703), medically insured (68%, n = 722), with no chronic medical conditions (90%, n = 955), and mostly Jordanians (84%, n = 887). Median age was 24 years old for the whole sample. The majority had, or were enrolled in, a higher education degree, either undergraduate (68%, n = 725) or postgraduate (16%, n = 165) [Table 1].

**Table 1:** Socio-demographic characteristics of participants.

Variable	N (%)
**Gender**	
Male	528 (49.8)
Female	533 (50.2)
**Age**	
< 24 years old	566 (53.4)
24–35 years old	306 (28.8)
> 35 years old	189 (17.8)
**Nationality**	
Jordanian	887 (83.6)
Non-Jordanian	174 (16.4)
**Marital status**	
Single	797 (75.1)
Married	247 (23.3)
Divorced	14 (1.3)
Widowed	3 (0.28)
**Level of education**	
None	6 (0.57)
Elementary	6 (0.57)
Secondary	111 (10.5)
Diploma	48 (4.5)
Bachelor	725 (68.3)
Masters, PhD, or equivalent	165 (15.5)
**Employment**	
Currently Employed	346 (32.6)
Unemployed	703 (66.3)
Retired	12 (1.1)
**Have health insurance**	
Yes	722 (68)
No	339 (32)
**Have a chronic medical condition**	
Yes	106 (10)
No	955 (90)

### Participants’ awareness of “clinical research”

**Fig 1:**
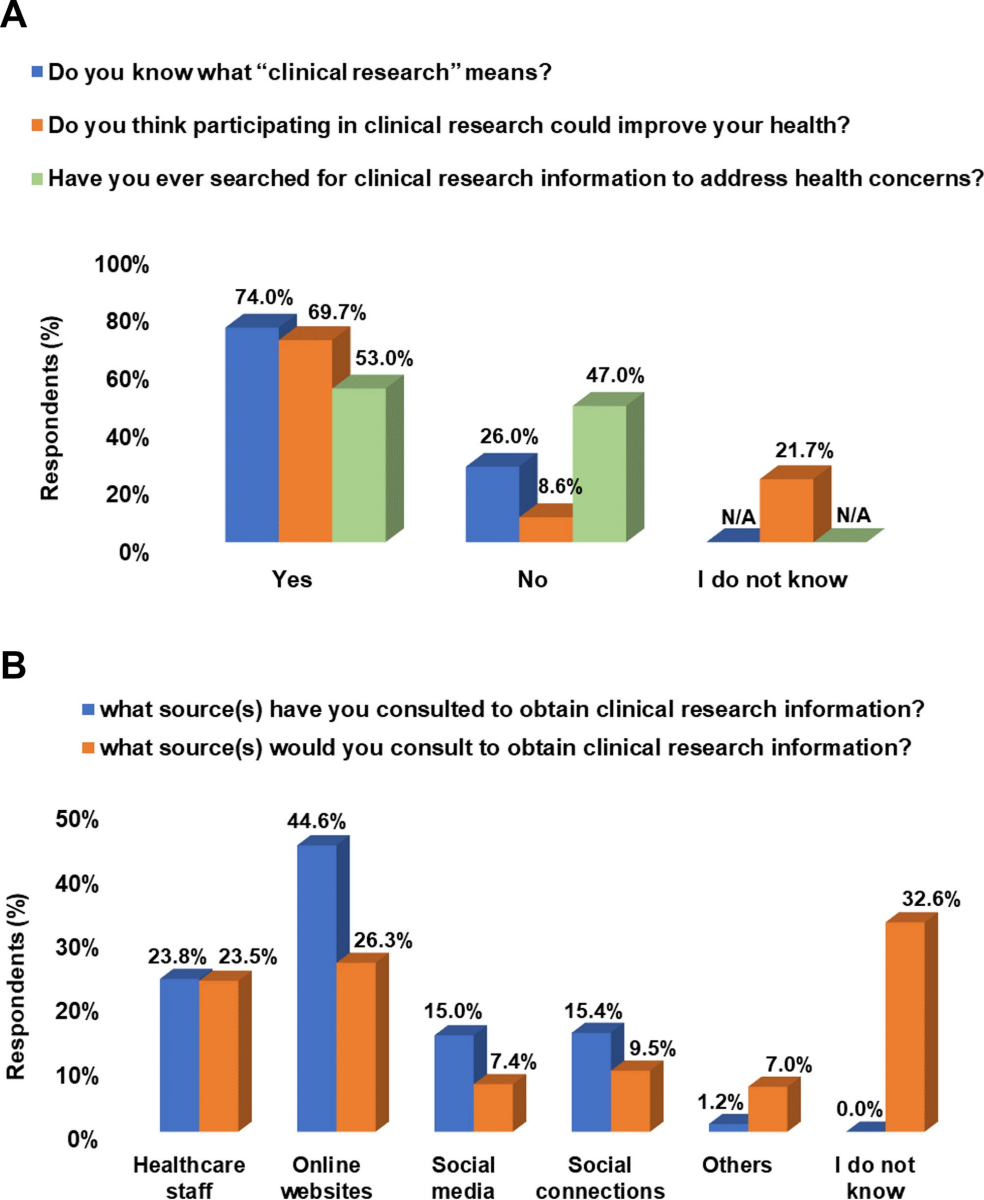
Assessment of participants’ awareness and understanding of clinical research. (A) Bar graph showing participants’ awareness of clinical research, its role in promoting health, and their clinical research information seeking behavior. (B) Clinical research information sources participants used or would use to address health-related concerns. All responses are reported as percentages out of the total (N = 1061).

The majority of participants reported being familiar with “Clinical Research” (74%, n = 785) as well as its role in improving general health (70%, n = 740). Most participants reported online websites and healthcare providers as the two main sources of CR information that they have previously consulted (45%, n = 473 and 24%, n = 252 respectively), or would like to consult (26%, n = 279 and 24%, n = 249 respectively) in the future. On the other hand, a relatively large proportion of the respondents (33%, n = 346) did not know which CR information source they should consult (Fig 1).

### Participants’ motivators and perceived barriers towards clinical research participation

**Fig 2:**
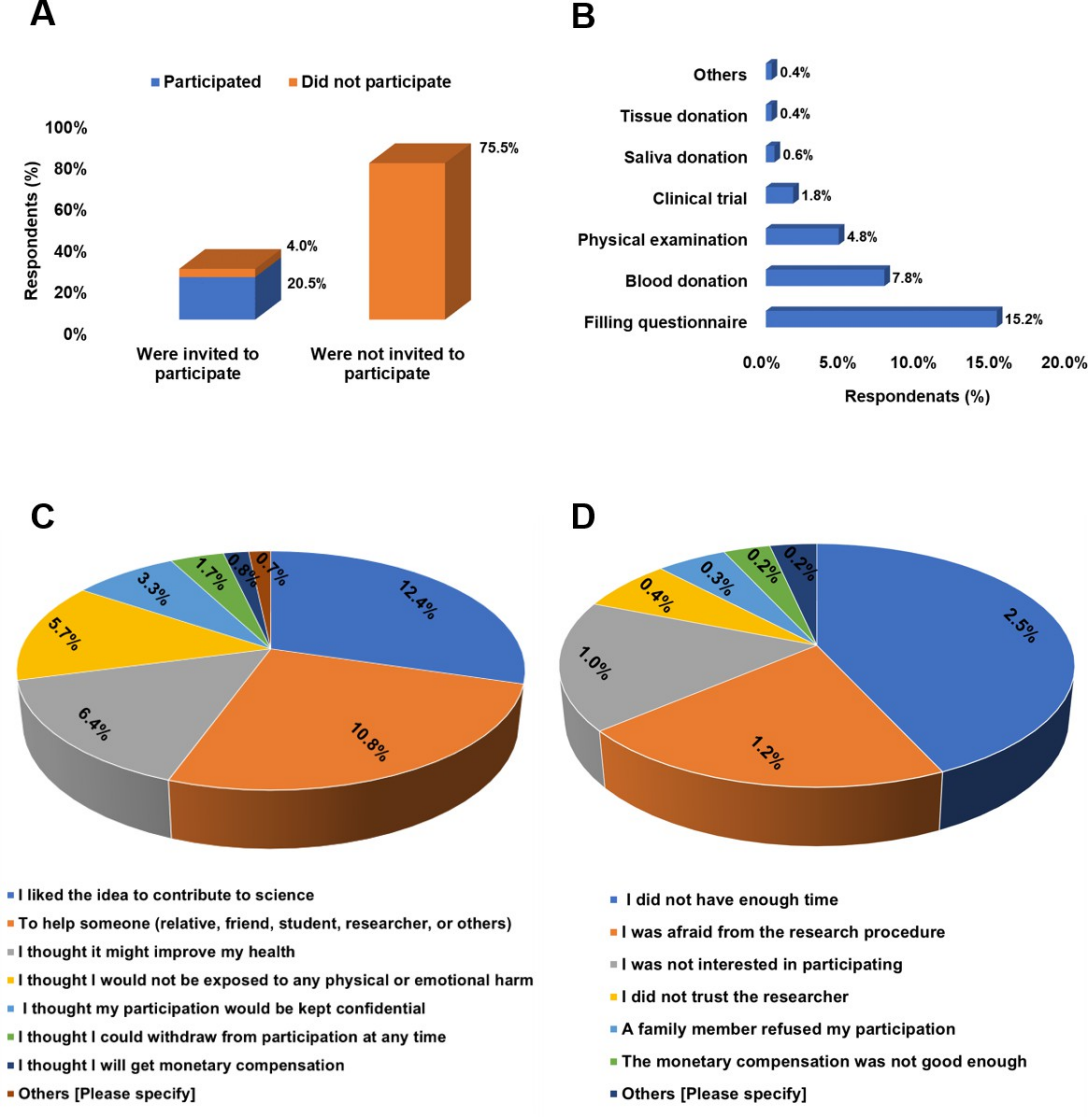
Assessing motivators and barriers towards prior participation in clinical research. (A) Bar graph showing percentage of participants who were invited to participate, including those who accepted and rejected participation. (B) Bar graph showing clinical research contributions offered by those who participated in clinical research. (C) Pie graph showing participation motivators for those who accepted to participate. (D) Pie graph showing participation barriers for those who rejected to participate. All responses are reported as percentages out of the total (N = 1061).

About 25% (n = 260) of participants had been previously invited to participate in CR, of which 21% (n = 218) had accepted, while 4% (n = 42) had declined. Contributing to science (12%, n = 132), helping others (1%, n = 7), and improving one’s health status (6%, n = 68) were the most cited motivators for participating in CR, while lack of enough time (2%, n = 26), fears regarding the research procedure (1%, n = 11), and lack of interest (1%, n = 10) were the main reasons for rejecting participation. Participants reported filling questionnaire surveys (15%, n = 161), blood sample donations (8%, n = 83), and participating in physical examinations (5%, n = 51) as their main contributions in the research, while clinical trial participation (2%, n = 19), donating saliva samples (0.6%, n = 6), and donating tissue samples (0.4%, n = 4) were their least cited prior contributions (Fig 2).

**Fig 3:**
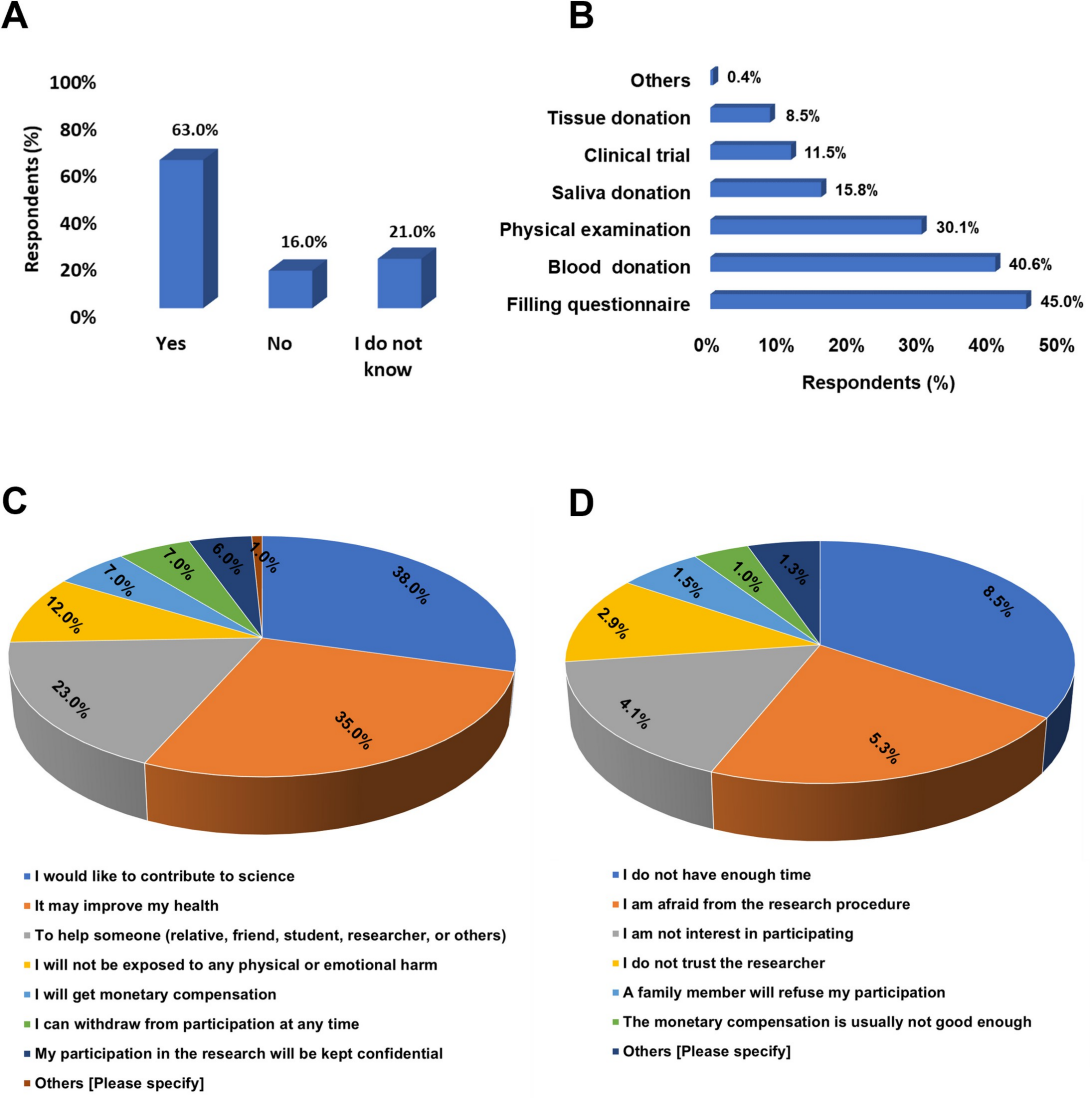
Assessing motivators and barriers towards future participation in clinical research. (A) Bar graph showing percentage of participants who are willing, not willing, or not sure to participate in future clinical research. (B) Bar graphic showing clinical research contributions that participants are willing to offer. (B) Pie graph showing perceived participation motivating factors for those who are willing to participate. (C) Pie graph showing perceived participation barriers for those who are not willing to participate. All responses are reported as percentages out of the total (N = 1061).

While most participants (63%, n = 672) were willing to participate in CR in the future, 21% (n = 218) were not sure if they would participate, and 16% (n = 171) refused future participation. Cross-tabulation of prior participation with willingness to participate in the future revealed a statistically significant difference between the two groups (P-value < 0.0001) [Table 2]. Contributing to science (38%, n 403), improving one’s health (35%, n = 371), and helping others (23%, n = 240) were the top motivators for those who were willing to participate, while lack of enough time (8%, n = 90), fears regarding the research procedure (5%, n = 56), and lack of interest (4%, n = 43) were the most cited reasons behind rejecting participation. Filling questionnaire surveys (45%, n = 477), blood sample donation (41%, n = 431), and participating in physical examination (30%, n = 319) were the main contributions the participants were interested in. Clinical trial participation (12%, n = 122), donating saliva samples (16%, n = 168), and donating tissue samples (8%, n = 90) were the contributions participants were least interested in (Fig 3). There was no statistically significant difference between prior research participation and participation interests (P-value = 0.209) [Table 2].

**Table 2:** Cross tabulation of prior clinical research participation with future participation, contribution interests, and attitudes towards clinical research conduct, confidentiality, and harm.

Question	Total, N (%)	Prior participation in clinical research, N (%)		
	1061 (100)	Yes, 218 (20.5)	No, 843 (79.5)	P value
**Would you accept to participate in clinical research in the future if you were invited to?**				
Yes	666 (62.7)	167 (15.7)	499 (47.0)	<0.0001**
No	167 (15.7)	20 (1.9)	147 (13.9)	
I do not know	228 (21.5)	31 (2.92)	197 (18.6)	
**What type of clinical research contribution(s) would you like to provide? (Please choose all that apply)**				
Filling questionnaire surveys	632 (59.6)	166 (15.6)	466 (43.9)	0.209
Donating blood samples	548 (51.6)	116 (10.9)	432 (40.7)	
Participating in physical examinations	372 (35)	89 (8.3)	283 (26.7)	
Participating in clinical trials	136 (12.8)	43 (4.1)	93 (8.8)	
Donating saliva samples	207 (19.5)	52 (4.9)	155 (14.6)	
Donating tissue samples	99 (9.3)	26 (2.5)	73 (6.9)	
Others [please specify]	10 (0.9)	2 (0.2)	8 (0.8)	
**How do you think clinical research is conducted?**				
I do not have an opinion	604 (56.9)	79 (7.4)	525 (49.5)	<0.0001**
Clinical research is conducted in a responsible and ethical manner	323 (30.4)	99 (9.3)	224 (21.1)	
Clinical research is conducted by unqualified personnel	93 (8.8)	28 (2.6)	65 (6.1)	
Clinical research is conducted in unethical manner	41 (3.9)	12 (1.1)	29 (2.7)	
**Do you think participating in clinical research exposes the participant to harm?**				
Strongly agree	42 (3.9)	6 (0.6)	36 (3.4)	<0.0001**
Agree	111 (10.4)	15 (1.4)	96 (9.0)	
I am not sure	518 (48.8)	83 (7.8)	435 (41.0)	
Disagree	283 (26.6)	82 (7.7)	201 (18.9)	
Strongly disagree	107 (10.1)	32 (3.0)	75 (7.1)	
**Do you think participating in clinical research maintains participant’s confidentiality?**				
Strongly agree	126 (15.2)	33 (3.1)	129 (12.1)	0.0109*
Agree	348 (32.8)	82 (7.7)	266 (25.1)	
I am not sure	470 (44.3)	78 (7.4)	392 (36.9)	
Disagree	61 (5.7)	17 (1.6)	44 (4.1)	
Strongly disagree	20 (1.9)	8 (0.8)	12 (1.1)	

*, ** refer to P value less than 0.05 and 0.0001, respectively.

### Participants’ attitudes towards clinical research

Almost half of the respondents (57%, n = 604) had no opinion when it comes to whether CR is conducted in a responsible and ethical manner. Although 30% (n = 323) believed that CR is conducted responsibly and ethically, about 4% (n = 41) and 9% (n = 93) thought it is conducted in an unethical manner or by unqualified personnel, respectively. On the other hand, a large percentage of participants were not sure if participating in CR maintains participants’ confidentiality (44%, n = 470) or exposes them to any form of harm (49%, n = 518). However, many participants either strongly agreed (15%, n = 126) or agreed (33%, n = 348) that CR maintains a high level of confidentiality and disagreed (27%, n = 283) that it imposes any form of harm to the participant. There was a statistically significant difference in responding to attitude questions between those who previously participated in clinical studies and those who did not (Table 2).

## Discussion

The increased prevalence and complexity of acute and chronic conditions and comorbidities, all have made it a difficult task to diagnose and treat illnesses and to provide optimal healthcare services in Jordan. For this reason, CR has become critically important in dealing with these challenges through informing clinical practice and improving healthcare services delivery. Because participants’ recruitment is an important step towards performing CR [3, 37], we therefore aimed in this population-based survey to investigate the public’s awareness of CR as well as their motivating factors and perceived barriers towards CR participation, and to discuss our findings in light of others from the region. A nearly equal number of males and females participated in the current survey. The majority were young to middle aged, well educated, unmarried, unemployed, medically insured Jordanians with no chronic clinical conditions. The study took place in Jordan, which is a country with a young population and has the highest literacy rate in the Arab world [9, 10, 38–41]. Besides, almost 70% of Jordanians are covered under formal health insurance that is paid for students and employees by academic and employing institutions, respectively [42, 43]. This may explain why most participants were Jordanians between 18–40 years of age (median age = 24 years), well educated, and medically insured, and because younger Jordanians have higher tendency to be unmarried [44], unemployed [45], and free of chronic diseases, it is unsurprising that these were the main socio-demographic characteristics of the participants.

Participating in CR implies being familiar with the concept of “clinical research” and its value in advancing science and promoting health [46–48]. Most participants (74%) reported being aware of “clinical research”. This awareness level is more than that reported by Lebanese participants in another study, where 45% were aware of this term [32]. About 70% of the current participants agreed that CR can promote general health. This percentage is relatively lower than a qualitative pilot study from Egypt where all participants believed that CR could improve their health condition [49], yet higher than a study from Saudi Arabia where only 51% of participants thought it can improve their general well-being [21]. In terms of using CR information for addressing health-related concerns, about 53% of our participants have sought CR information before. Online websites were the main source of CR information for those participants, followed by healthcare staff. In fact, these two were also the main sources for those who were willing to search for CR information. These findings are not surprising as there are multiple trustworthy online information sources that are readily accessible in Jordan. Indeed, in the last decade, the internet has become an important medium for shopping, socializing, and obtaining reliable information. Additionally, due to its low cost, comprehensibility, reliability, and ease of accessibility, it has become, for many, the preferred source of information. Notably, our assessment of participants’ awareness towards CR revealed that about 26% did not understand the term “clinical research”, 21% did not have an opinion regarding CR benefits on health, and nearly 33% did not know which source they should use to search for information. We identify those participants as potential targets for future campaigns that aim to raise awareness towards CR key elements and its trusted sources of information.

In terms of CR participation, only a small number of participants (25%) have received prior invitations to participate in CR, of which 21% and 4% accepted and rejected participation, respectively, resulting in a calculated rejection rate of 16%. This suggests that the relatively high awareness of CR reported in this study by participants is not solely as a result of prior participation, but rather associated with other factors such as the high literacy rate (P-value < 0.0001), although those who previously participated were relatively more aware of CR than those who did not (P-value < 0.0001). This may also suggest that most recruitments in the country take place in clinical settings, leaving the general public out of the recruitment equation most of the time. Therefore, modifications to the local recruitment strategies to outreach this potentially eligible population is highly recommended to more accurately reflect the research findings [50]. In addition, the fact that a large percentage (63%) of the participants showed willingness to participate in future CR further supports this recommendation. Similar findings were observed in Qatar and Saudi Arabia, where 63% and 74% of participants showed positive attitudes towards participation [20, 21]. On the other hand, around 16% of our participants rejected future participation in CR, which is a similar rejection rate reported by subjects who were previously invited to participate. Nonetheless, those who previously participated in CR were more willing to participate in the future compared to those who did not (P < 0.0001). In fact, the influence of previous participation in promoting positive attitude towards future participation has also been reported by Al-Tannir et al. (2016) in Saudi Arabia [21]. In our study, those who did not know if they would accept or reject future participation (21%) would probably want to know more of the type of CR and the potential risks and benefits associated with it before they can decide on participation [21, 51].

Assessing motivators for CR participation revealed that contributing to science and benefiting others were the two major motivating factors for both prior and future participations in CR. Even though Jordan is a developing country (https://datahelpdesk.worldbank.org/knowledgebase/articles/906519-world-bank-country-and-lending-groups) with a high unemployment rate (https://www.worldbank.org/en/country/jordan/overview), financial incentive was not among the top four motivators and was the least cited barrier for CR participation. Although monetary compensation [52] and self-benefits [49] have been reported in the literature as major facilitators to CR participation, our findings are more in agreement with those from high-income countries in the region such as Saudi Arabia, UAE, and Qatar [20, 21, 24]. One may argue that this sense of altruism in the Jordanian population has its moral roots, theological origin, or both, and manifested in welcoming a large number of refugees from conflict areas and may provide a sense of usefulness and satisfaction to the participants [11, 53].

On the other hand, time constraints, worries from the research procedure, and lack of interest were the main cited barriers to CR participation. Time constraint as a barrier was also reported from the non-national Qatari population [20]. It could be the case that in a developing country such as Jordan, most of the general public have to work long hours to make a living. This is in addition to the increased unemployment rate resulting from the recent population expansion; all of which make time a valuable asset to Jordanians [54]. Therefore, making the recruitment process as smooth and well organized as possible will most likely enhance public’s participation. Additionally, fear regarding the research procedure reported in our study is not an uncommon hindrance to CR participation [4, 55, 56]. Such fear was reported to stem from perceived risks of interventions, their potential side effects, and/or confidentiality issues [57–59]. In this regard, improvements to the ethics and regulatory review procedures, improvements to the informed consent process, as well as efficient researcher-participant communication all have been reported to greatly enhance the recruitment procedure [54, 60–63]. The researcher can assure participants that the study was carefully designed to not cause harm to the participants and was approved by a research ethics committee which ensures that the study is conducted in compliance with ethical standards, and that their participation in the study will not be disclosed or impact their care or career [51]. The third most cited barrier to participation was lack of interest. Lack of interest in CR participation has been attributed to several reasons such as lack of health literacy and numeracy [64], lack of self-benefits [65], social/cultural constraints [66], unpleasant/unsatisfactory experience with prior participation [67], and/or lack of feedback from the researcher at the end of the study [68]. Most of these issues contributing to lack of interest in participation can be overcome through effective researcher-participant communication prior to, during, and after recruitment has taken place and will most likely enhance the recruitment process and participants retention [69, 70].

Filling questionnaires, donating blood samples, and physical exam participation were the most highlighted previous contributions as well as the contributions participants were most interested-in in the future. Similar findings were observed in Qatar [20]. These results suggest that CR in Jordan that recruits a sample from the general population mostly demands these types of contributions. On the other hand, clinical trials, donating saliva samples, and donating tissue samples were the least cited prior contributions and the ones that participants were least interested in. Notably, prior participation in CR did not affect contribution interests as there was not a statistically significant difference in contribution interests between those who previously participated and those who did not. This suggests that future contribution interests may not be directly related to participants’ prior experience with these CR contributions but rather the perceived degree of convenience and safety associated with them. Indeed, participants tend to be reluctant to participate in procedures that are largely invasive or that they perceive to involve a high degree of inconvenience [21, 71].

Assessing participants’ attitudes revealed that many participants did not have a clear opinion towards the ethical conduct (57%), confidentiality (44%), and harm (49%) associated with CR. The lack of opinion to these topics is not mainly arising from low education level or awareness as most participants were well-educated and aware of CR, but rather stems from a lack of prior experience in CR participation as the majority (80%) have not participated in a CR before. In support of this, cross-tabulation of prior participation with attitudes towards CR ethical conduct, confidentiality, and harm revealed a statistically significant difference (P values < 0.0001, <0.0001, and < 0.0109, respectively) [Table 2]. We also envisage this lack of opinion to explain, at least in part, why 21% of participants were not sure if they would accept or decline future CR participation invites. More importantly, these findings suggest CR participation as an effective strategy to promote positive attitudes towards CR. In addition, although not assessed in the current study, several studies from Jordan highlighted ethical challenges in CR which include the IRB review process, the informed consent process, and lack of ethical training for researchers [72–77]. As a result, implementing research ethics guidelines as well as training in the responsible conduct of research would seem imperative to overcome those challenges [77, 78].

## Limitations

The current study comes with some limitations that can be mitigated through future research. First, the study did not assess participants’ knowledge of key elements of CR, what it entails, or their misconceptions, rather, it assessed their awareness of and familiarity with the “clinical research” terminology. Although, the definition of CR and its scope were briefly explained to study participants, it is still a possibility that some study participants did not distinguish between certain closely related types of research such as CR versus public health research. Moreover, several other facilitators and barriers that can affect CR participation were not covered in the current study. For that matter, we only addressed the top cited motivators and barriers reported from previous studies in the region. Additionally, although the health care system in Jordan was greatly affected by the inflex of refugees from neighboring countries of conflict, this study did not assess the refugees’ attitudes towards CR participation. Future studies that address refugees’ knowledge of and attitudes towards CR will be of great value.

Moreover, although our study recruited participants from different areas of different cities, our sampling method, by definition, remains convenience sampling, and the ability to generalize from convenience sampling remains limited compared to random sampling. As a potential consequence of convenient sampling, the current study mostly recruited young participants, mainly excluding those aged 40 or above (Table 1). It could be argued that CR conducted in Jordan often recruits from populations with particular diseases, who in turn tend to be older in age. Therefore, this could be another explanation for the previously low CR participation rate of the respondents. Additionally, although Current results indicate that about 16% of the participants were non-Jordanian. The questionnaire used in the current study, however, did not inquire about the nationality of this group.

## Conclusion

Clinical research participation of the public in Jordan needs improvement. The previously low CR participation rate of our sample reported in the current study originates from a lack of outreach to the public rather than a lack in their willingness to participate. Most of the Jordanian population are aware of CR and its role in promoting health. This awareness was associated with the high literacy rate of Jordanians and may reflect a research culture in the country. However, many participants lack or have negative attitudes when it comes to some aspects of CR such as ethical conduct, confidentiality, and potential harm. This comes largely because of lack of prior experience in CR participation. Therefore, modifying recruitment strategies to include more of the public in clinical studies in light of participation facilitators and barriers identified in this study is highly recommended in the early planning of CR to first reflect and generalize the findings of these studies to the general population and second to promote positive attitudes to CR.

## Supporting information

S1 File The English version of the questionnaire. (DOCX)

S1 Dataset (CSV)
