# Supplementary material for: Motivators and barriers towards clinical research participation: A population-based survey from an Arab MENA country
Source: PLoS One. 2022 Jun 24;17(6):e0270300. doi: 10.1371/journal.pone.0270300 (PMC9231817; doi:10.1371/journal.pone.0270300)
Supplement: S1 File — (DOCX) [file pone.0270300.s001.docx]

Dear participant,

We invite you to fill in this questionnaire which is part of a research study that assesses clinical research participation in Jordan. Clinical research is a type of research that aims to generate valuable knowledge for understanding, diagnosing, preventing and treating diseases. If you agree to participate, then please answer the questions objectively and be assured that all views/opinions will be treated confidentially and will only be used for the purpose of this study.

Many thanks in advance!

**Questionnaire**

**I. Socio-demographic characteristics**

| **Gender** | - Male - Female |
| --- | --- |
| **Age (years)** |  |
| **Nationality** | - Jordanian - Non-Jordanian |
| **Marital status** | - Unmarried - Married - Divorced - Widowed |
| **Level of education** | - None - Elementary - Secondary - Diploma - Undergraduate - Postgraduate |
| **Employment** | - Currently Employed - Unemployed - Retired |
| **Do you have health insurance?** | - Yes - No |
| **Do you have any chronic medical conditions?** | - Yes - No |

**II. Assessing awareness of clinical research**

**Do you know what “clinical research” means?**

- Yes
- No

**Have you ever searched for clinical research information to address health concerns?**

- Yes
- No

**If yes, what source(s) have you consulted to get the btain clinical research information? (Please choose all that apply)**

- Healthcare staff (medical doctor, nurse, pharmacist, or others)
- Online (trusted website, specialized governmental websites, specialized non-governmental websites)
- Social media (Facebook, Telegram, or others)
- Personal connections (relative, friend, colleague)
- Others [Please specify]

**If no, what source(s) would you consult to obtain clinical research information? (Please choose all that apply)**

- Healthcare staff (medical doctor, nurse, pharmacist, or others)
- Online websites (trusted website, specialized governmental websites, specialized non-governmental websites)
- Social media (Facebook, Telegram, or others)
- Social connections (relative, friend, colleague)
- Others [Please specify]

**Do you think participating in clinical research could improve your general health?**

- Yes
- No
- I do not know

**III. Assessing motivators and barriers towards clinical research participation**

**Have you ever been invited to participate in a clinical research?**

- Yes
- No

**If yes, have you accepted the invitation to participate?**

- Yes
- No

**If yes, why have you accepted to participate? (Please choose all that apply)**

- I thought it might improve my health
- I thought I will get monetary compensation
- I thought my participation would be kept confidential
- I thought I would not be exposed to any physical or emotional harm
- I thought I could withdraw from participation at any time
- I liked the idea to contribute to science
- To help someone (relative, friend, student, researcher, or others)
- Others [Please specify]

**If yes, then what was your contribution(s) in the clinical research you have participated in? (Please choose all that apply)**

- Filling questionnaire surveys
- Participating in physical examinations
- Donating blood samples
- Donating saliva samples
- Donating tissue samples
- Participating in clinical trials
- Others [Please specify]

**If you refused the invitation to participate in a clinical research, then why? (Please choose all that apply)**

- I did not have enough time
- I was afraid from the research procedure
- I was not interested in participating
- I did not trust the researcher
- A family member refused my participation
- The monetary compensation was not good enough
- Others [Please specify]

**Would you accept to participate in a clinical research in the future if you were invited to?**

- Yes
- No
- I do not know

**If yes, then why would you accept to participate? (Please choose all that apply)**

- It may improve my health
- I will get monetary compensation
- My participation in the research will be kept confidential
- I will not be exposed to any physical or emotional harm
- I can withdraw from participation at any time
- I would like to contribute to science
- To help someone (relative, friend, student, researcher, or others)
- Others [Please specify]

**If yest, then what type of clinical research contribution(s) would you like to provide? (Please choose all that apply)**

- Filling questionnaire surveys
- Participating in physical examinations
- Donating blood samples
- Donating saliva samples
- Donating tissue samples
- Participating in clinical trials
- Others [please specify]

**If you would refuse the invitation to participate in a clinical research, then why? (Please choose all that apply)**

- I do not have enough time
- I am afraid from the research procedure
- I am not interested in participating
- I do not trust the researcher
- A family member will refuse my participation
- The monetary compensation is usually not good enough
- Others [Please specify]

**IV. Assessing attitudes towards clinical research**

**How do you think clinical research is conducted?**

- Clinical research is conducted in a responsible and ethical manner
- Clinical research is conducted by unqualified personnel
- Clinical research is conducted in unethical manner
- I do not have an opinion

**Do you think participating in clinical research exposes the participant to harm?**

- Strongly agree
- Agree
- I am not sure
- Disagree
- Strongly disagree

**Do you think participating in clinical research maintains participants’ confidentiality?**

- Strongly agree
- Agree
- I am not sure
- Disagree
- Strongly disagree
